# Misoprostol Inhibits Equine Neutrophil Adhesion, Migration, and Respiratory Burst in an *In Vitro* Model of Inflammation

**DOI:** 10.3389/fvets.2017.00159

**Published:** 2017-09-28

**Authors:** Emily Medlin Martin, Rebecca Louise Till, Mary Katherine Sheats, Samuel L. Jones

**Affiliations:** ^1^Department of Clinical Sciences, College of Veterinary Medicine, North Carolina State University, Raleigh, NC, United States; ^2^College of Veterinary Medicine, Comparative Medicine Institute, North Carolina State University, Raleigh, NC, United States

**Keywords:** horse, leukocyte, chemotaxis, reactive oxygen species, inflammation, anti-inflammatory, NSAID

## Abstract

In many equine inflammatory disease states, neutrophil activities, such as adhesion, migration, and reactive oxygen species (ROS) production become dysregulated. Dysregulated neutrophil activation causes tissue damage in horses with asthma, colitis, laminitis, and gastric glandular disease. Non-steroidal anti-inflammatory drugs do not adequately inhibit neutrophil inflammatory functions and can lead to dangerous adverse effects. Therefore, novel therapies that target mechanisms of neutrophil-mediated tissue damage are needed. One potential neutrophil-targeting therapeutic is the PGE_1_ analog, misoprostol. Misoprostol is a gastroprotectant that induces intracellular formation of the secondary messenger molecule cyclic AMP (cAMP), which has been shown to have anti-inflammatory effects on neutrophils. Misoprostol is currently used in horses to treat NSAID-induced gastrointestinal injury; however, its effects on equine neutrophils have not been determined. We hypothesized that treatment of equine neutrophils with misoprostol would inhibit equine neutrophil adhesion, migration, and ROS production, *in vitro*. We tested this hypothesis using isolated equine peripheral blood neutrophils collected from 12 healthy adult teaching/research horses of mixed breed and gender. The effect of misoprostol treatment on adhesion, migration, and respiratory burst of equine neutrophils was evaluated *via* fluorescence-based adhesion and chemotaxis assays, and luminol-enhanced chemiluminescence, respectively. Neutrophils were pretreated with varying concentrations of misoprostol, vehicle, or appropriate functional inhibitory controls prior to stimulation with LTB_4_, CXCL8, PAF, lipopolysaccharide (LPS) or immune complex (IC). This study revealed that misoprostol pretreatment significantly inhibited LTB_4_-induced adhesion, LTB_4_-, CXCL8-, and PAF-induced chemotaxis, and LPS-, IC-, and PMA-induced ROS production in a concentration-dependent manner. This data indicate that misoprostol-targeting of E-prostanoid (EP) receptors potently inhibits equine neutrophil effector functions *in vitro*. Additional studies are indicated to further elucidate the role of EP receptors in regulating neutrophil function. Overall, our results suggest misoprostol may hold promise as a novel anti-inflammatory therapeutic in the horse.

## Introduction

Neutrophils provide a first-line defense against all types of tissue insult, including invading bacterial pathogens, and sterile tissue injury in both humans and horses. Upon infection, neutrophils move from the vasculature into areas of tissue inflammation by an intricate mechanism of recruitment and activation. This process includes adhesion, crawling, extravasation, interstitial tissue migration, and culminates in the release of bactericidal products such as reactive oxygen species (ROS) and antibacterial proteins (1). While these steps are necessary to defend the host against pathogens, dysregulated or overabundant neutrophil responses elicit substantial host tissue injury (2, 3). Indeed, neutrophils have been implicated in the pathogenesis of many devastating disorders in horses including laminitis (4, 5), heaves (6, 7), and gastrointestinal ischemia-reperfusion injury (8). Horses diagnosed with these conditions could potentially benefit from therapeutics that prevent or minimize excessive neutrophil activation.

Currently, therapies designed to inhibit neutrophilic inflammation in humans and animals are limited (9), and novel targets for neutrophil inhibition must be identified. One potential molecular target known to regulate neutrophil functions is cyclic AMP (cAMP). cAMP is a ubiquitously produced second messenger molecule that is generated intracellularly through neutrophil G-protein coupled receptor (GPCR) signaling. Inflammatory ligands such as cytokines bind to GPCRs and activate intracellular adenylate cyclase (AC), which catalyzes the cyclization of AMP to form cAMP. cAMP regulation is essential for neutrophil functions including adhesion (10, 11), chemotaxis (12), and production of ROS (11, 13, 14).

Naturally occurring cAMP-elevating agents include E-type prostaglandins (PGEs) PGE_1_ and PGE_2_. Binding of PGEs to E-prostanoid (EP) 2 and EP4 receptors increases intracellular cAMP and attenuates multiple neutrophil functions *in vitro* (15–20). Unfortunately, clinical use of prostaglandins is limited because they are unstable and have poor oral bioavailability. One PGE analog that is both stable and well absorbed orally is misoprostol (21). Misoprostol is an EP2, EP3, and EP4 receptor agonist that increases intracellular cAMP and is FDA-approved to treat NSAID-induced ulceration in humans (21–23). In horses, misoprostol has been shown to decrease gastric acid secretion, increase recovery of ischemia-injured equine jejunum, and is currently used to treat NSAID-induced colitis and ulceration (24–26). The anti-inflammatory properties of misoprostol, however, have yet to be studied in equine neutrophils. Therefore, our goal was to evaluate misoprostol as a novel anti-inflammatory therapeutic in equine neutrophils. We hypothesized that the PGE_1_ analog misoprostol would inhibit proinflammatory functions of stimulated equine neutrophils *in vitro*. This study is the first to demonstrate that misoprostol pretreatment attenuates equine neutrophil adhesion, chemotaxis, and ROS production in a concentration-dependent manner.

## Materials and Methods

### Experimental Reagents

Lipopolysaccharide (LPS) from *E. coli* 055:B5, phorbol 12-myristate 13-acetate (PMA), CXCL8, dibutyryl cyclic AMP (db-cAMP), wortmannin, staurosporine, bovine serum albumin (BSA), and anti-BSA antibody were from Sigma Aldrich (St. Louis, MO, USA); heat-inactivated fetal bovine serum (FBS) was from Gemini-Bioproducts (West Sacramento, CA, USA); misoprostol, LTB_4_, and PAF were from Cayman Chemical (Ann Arbor, MI, USA); equine recombinant granulocyte-monocyte colony-stimulating factor (GM-CSF) was from Kingfisher Biotech (Saint Paul, MN, USA); and Hank’s balanced salt solution (HBSS) was from Thermo Fisher Scientific (Grand Island, NY, USA).

### Equine Donors and Neutrophil Isolation

All experiments were approved by the Institutional Animal Care and Use Committee at North Carolina State University (NCSU). Horses included in this study were part of the NCSU Teaching Animal Unit herd, 5–15 years of age, and of mixed breed and gender. All horses were deemed healthy upon physical examination of a board-certified equine internal medicine specialist and were housed under similar conditions and did not receive any medications for the duration of the study.

Neutrophils were isolated from equine whole blood by density-gradient centrifugation as previously described (27). Briefly, 30–60 cc of heparinized equine whole blood was collected *via* jugular venipuncture. Whole blood was placed into sterile conical tubes for 1 h at room temperature to allow erythrocytes to settle out of suspension. The leukocyte-rich plasma (supernatant) was layered onto Ficoll-Paque Plus (GE Healthcare, Sweden) at a 2:1 ratio. Cells were centrifuged and erythrocyte contamination was removed from the neutrophil pellet *via* 1-min hypotonic lysis.

### Misoprostol Pretreatment

Neutrophils were pretreated with indicated concentrations of misoprostol, db-cAMP, wortmannin, staurosporine, or vehicle for each inhibitor, for 30 min at 37°C. Cell viability was evaluated before and after pretreatment using trypan blue exclusion and was routinely >98%.

### Neutrophil Adhesion

Equine neutrophil adhesion methods have been optimized in our lab previously (27). Neutrophils were resuspended to a concentration of 1 × 10^7^ cells per ml in HBSS. 2 µg/ml of the fluorescent dye calcein AM (Anaspec, Fremont, CA, USA) was added to cells and incubated in the dark at room temperature for 30 min. Following calcein AM-labeling, cells were resuspended at 2.0 × 10^6^ in HBSS supplemented with 1 mM Ca^2+^, 1 mM Mg^2+^, and 2% FBS. For immune complex (IC)-induced adhesion, Immulon2HB plates (Thermo Fisher Scientific) were coated with 10 µg BSA overnight at 4°C and then incubated at 37°C for 2 h with 5 µg of anti-BSA antibody. 1 × 10^5^ cells were plated per well and incubated for 30 min at 37°C. Wells that were not coated with anti-BSA antibodies served as unstimulated controls. For LTB_4_- and PMA-induced adhesion, plates were coated overnight with 5% FBS at 4°C. 1 × 10^5^ cells were plated in each well and allowed to rest at 37°C for 10 min before addition of 10 ng/ml PMA (or 1 × 10^−5^ % DMSO vehicle) or 10 nM LTB_4_ (or 3 × 10^−3^ % ethanol vehicle). Cells were incubated at 37°C for 30 min with PMA or 75 s with LTB_4_.

Following incubation, fluorescence readings were obtained using an fMax plate reader (485 nm excitation, 530 nm emission) to obtain initial fluorescence, and then again after the second (LTB_4_) and third (IC and PMA) wash. Percent adhesion was calculated as the difference between the initial and final fluorescence readings in each well. Treatment conditions were performed in triplicate on each plate.

### Neutrophil Chemotaxis

Equine neutrophil chemotaxis methods have been optimized in our lab previously (27, 28). Neutrophils were labeled with calcein AM and resuspended in media as described for adhesion experiments. Neuroprobe disposable ChemoTx Systems (Neuroprobe, Gaithersburg, MD, USA) with 3-µm pore size and polycarbonate track-etch filters were used. Cell media containing chemoattractant or vehicle was added to the bottom chamber of each well. Chemoattractants included 10 nM LTB_4_, 10 nM PAF, 100 ng/ml CXCL8, and vehicle for each chemoattractant (3 × 10^−3^ % ethanol for LTB_4_ and PAF, HBSS for CXCL8). 100% migration control wells were prepared by adding 1 × 10^4^ calcein AM-labeled cells to the bottom chamber of three wells. Porous filters were placed over the bottom chambers so that contact between filter and chemoattractant or control media was established. 1 × 10^4^ misoprostol- or control-treated, calcein AM-labeled neutrophils were then added to the top portion of the membrance. Plates were incubated for 1 h at 37°C to allow directed cell migration into the bottom chambers (27). Following incubation, non-migrated cells were removed from the top of the filters and EDTA was added for 10 min at room temperature to detach cells remaining within the membrane. EDTA was removed and fluorescence of the bottom well was measured using an fMax plate reader as described above. Percent cell migration was determined by percent fluorescence of wells in each treatment group compared to the 100% migration control wells. Treatment conditions were performed in triplicate on each plate.

### Neutrophil ROS Production

Production of ROS was measured using luminol-enhanced chemiluminescence as previously optimized for equine neutrophils (29). Cells were plated on sterile, white, 96-well high-binding plates (Sigma), which were coated with 5% FBS (for LPS- and PMA-mediated respiratory burst) or 5 μg/well IC (for IC-mediated respiratory burst) as described above for adhesion experiments. Neutrophils were then stimulated using three different experimental protocols: (1) priming for 30 min with 1 ng/ml GM-CSF, followed by stimulation with 100 ng/ml LPS (or PBS vehicle); (2) 100 ng/ml PMA (or 0.01% DMSO vehicle); or (3) 5 µg/well immobilized IC (or no IC unstimulated control). 1 mM luminol was added to each well, and luminesce was measured every 5 min using a Fluoroskan Ascent FL Microplate Fluorometer and Luminometer (Thermo Scientific).

As this assay had not yet been attempted in our lab, preliminary experiments were conducted prior to data collection to determine the optimal number of neutrophils to be used for each stimulant. A standard curve was created by plotting neutrophil cell numbers versus raw luminescence values. From this curve, neutrophil quantities that fell on the linear portion of the curve were selected for analysis (data not shown). In accordance with this preliminary data, neutrophils were resuspended to achieve a final concentration of 3 × 10^5^ cells/well (IC), 2 × 10^5^ cells per well (LPS), or 1 × 10^5^ cells/well (PMA) in HBSS supplemented with 10 µM Ca^2+^, 10 µM Mg^2+^, and 2% FBS.

A series of kinetics studies were completed to determine the time of maximal significant ROS production in response to each stimulant. Based on these results, time points selected were 35 min for LPS, 40 min for PMA, and 55 min for IC. The effect of misoprostol pretreatment on stimulated ROS production was then evaluated at those time points. ROS production in misoprostol-pretreated cells was determined as a percentage of stimulated cells. Treatment conditions were performed in triplicate on each plate for both kinetics and experimental studies.

### Statistical Analysis

Data were analyzed using SigmaPlot (Systat Software, San Jose, CA, USA). All data were normally distributed (Shapiro–Wilk test) and are presented as mean ± SEM. Significant differences between treatments were determined with One-Way Repeated Measures Analysis of Variance (One-Way RM ANOVA) with Holm–Sidak multiple comparisons *post hoc* testing, or two-tailed *t*-test, where appropriate. ROS production from one horse was considered a significant outlier *via* the ESD (extreme studentized deviate) method and was excluded from analysis (α = 0.05). A *p*-value < 0.05 was considered statistically significant.

## Results

### Misoprostol Pretreatment Inhibits LTB_4_- but Not PMA- or IC-Induced Equine Neutrophil Adhesion

We hypothesized that the cAMP-elevating agent misoprostol would decrease equine neutrophil adhesion, and that a cell-permeant cAMP analog (db-cAMP) would serve as a model for increased intracellular cAMP and thus a positive control for inhibition of adhesion in our experiments. To examine the effects of misoprostol on varying degrees of β_2_ integrin binding, we stimulated equine neutrophil adhesion with the GPCR agonist LTB_4_, the FcγR agonist immune complexes (IC), or a direct PKC agonist phorbol 12-myristate 13-acetate (PMA).

**Figure 1:**
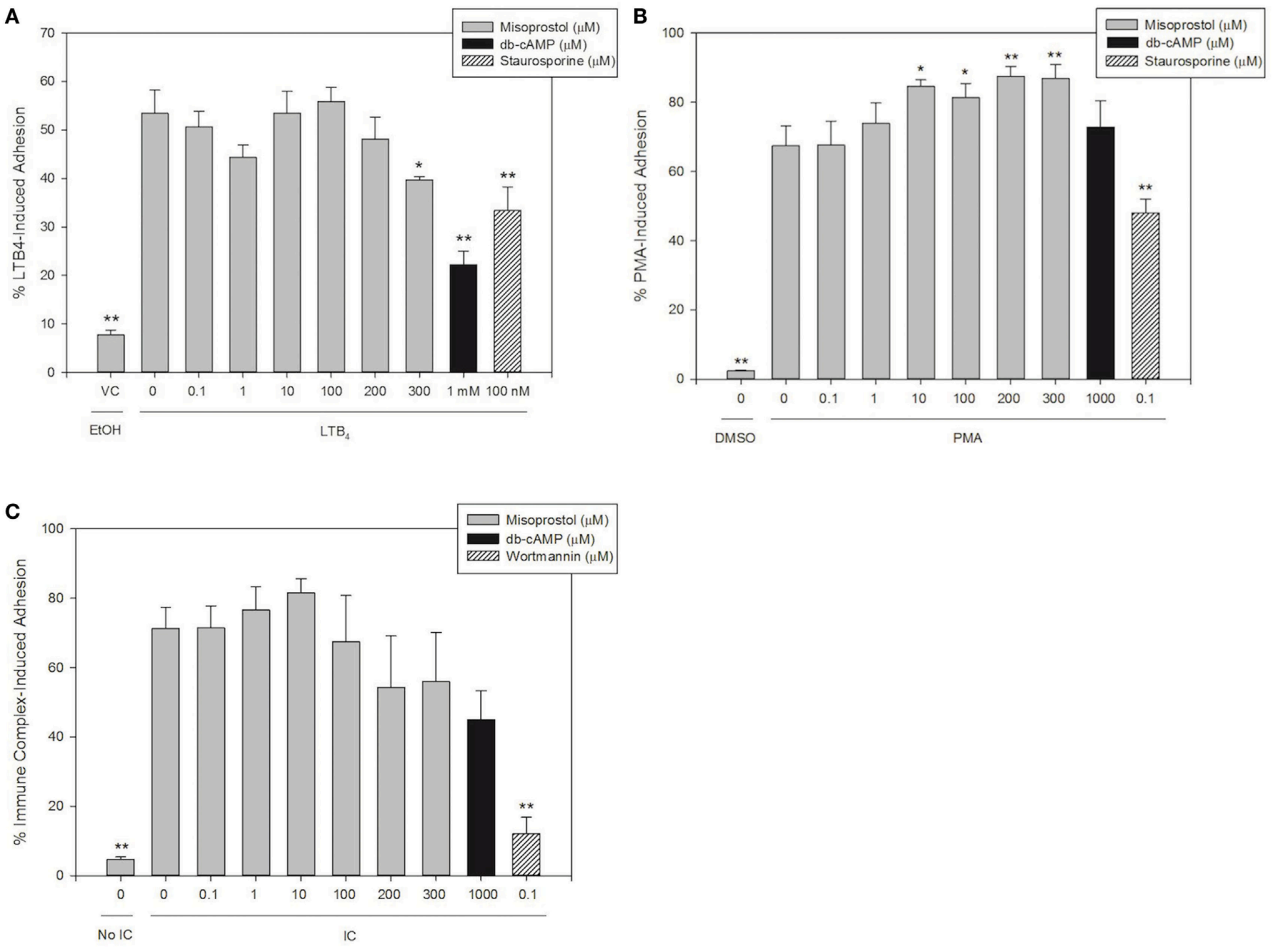
Misoprostol pretreatment inhibits LTB_4_- but not immune complex (IC)- or PMA-induced equine neutrophil adhesion. Calcein AM-labeled equine neutrophils were pretreated with varying concentrations of misoprostol, dibutyryl cyclic-AMP (db-cAMP), or the vehicle control (VC) for each inhibitor (Hank’s balanced salt solution). Preincubation with known inhibitors of neutrophil adhesion—wortmannin (for IC) or staurosporine (for LTB_4_ and PMA)—were utilized as a positive control for inhibition. Neutrophils were stimulated with the following stimulants or vehicles: **(A)** 10 nM LTB_4_ (or EtOH vehicle), **(B)** 100 ng/ml PMA (or DMSO vehicle), **(C)** 5 µg immobilized IC (or 5% BSA vehicle). Cells were stimulated with LTB_4_ for 75 s, or PMA and IC for 30 min. Initial fluorescence readings were taken prior to removal of non-adherent neutrophils *via* multiple washing steps including two washes for LTB_4_, and three washes for IC and PMA. Percent adhesion was calculated as the final fluorescence reading versus the initial fluorescence reading in each well. Data are expressed as mean% adhesion ± SEM. **p < 0.001 and *p < 0.05 indicate significant difference from stimulated cells pretreated with misoprostol vehicle *via* One-Way RM ANOVA; *n* = 3.

Our results show that 53.5% of LTB_4_-stimulated neutrophils adhered to FBS-coated plates. Misoprostol pretreatment (300 µM) significantly inhibited LTB_4_-stimulated neutrophil adhesion to 39.8%, and only 22.2% of LTB_4_-stimulated neutrophils pretreated with db-cAMP were adherent in our assay (Figure 1A). The PKC inhibitor staurosporine significantly attenuated LTB_4_-stimulated neutrophil adhesion to 33.4%, supporting a role for PKC in cytokine induced equine neutrophil adhesion (Figure 1A). In contrast, the phosphatidylinositol-3 kinase (PI3K)-inhibitor wortmannin had no effect on LTB_4_-stimulated adhesion of equine neutrophils.

Immune complex stimulated 71.3% adhesion of equine neutrophils in our assay. Interestingly, pretreatment of cells with misoprostol and db-cAMP had no significant effect on IC-induced adhesion (Figure 1B). Wortmannin pretreatment significantly inhibited IC-induced adhesion to 12.1%.

PMA stimulated approximately 67.5% of equine neutrophils to become adherent. Our results show that db-cAMP had no significant effect on PMA-mediated neutrophil adhesion; however, misoprostol pretreatment significantly enhanced PMA-induced adhesion to a maximum of 87.5%. Neutrophil pretreatment with the PKC inhibitor staurosporine was utilized as a positive control for inhibition in this assay and significantly inhibited PMA-induced equine neutrophil adhesion to 48.0% (Figure 1C).

### Misoprostol Pretreatment Inhibits Equine Neutrophil Migration toward LTB_4_, CXCL8, and PAF

To our knowledge, there are no previous reports on the effect of the PGE_1_ analog misoprostol on the chemotaxis of neutrophils from any species. In this study, we stimulated equine neutrophils to migrate utilizing CXCL8, LTB_4_, and PAF in order to investigate the effects of misoprostol pretreatment on equine neutrophil chemotaxis.

**Figure 2:**
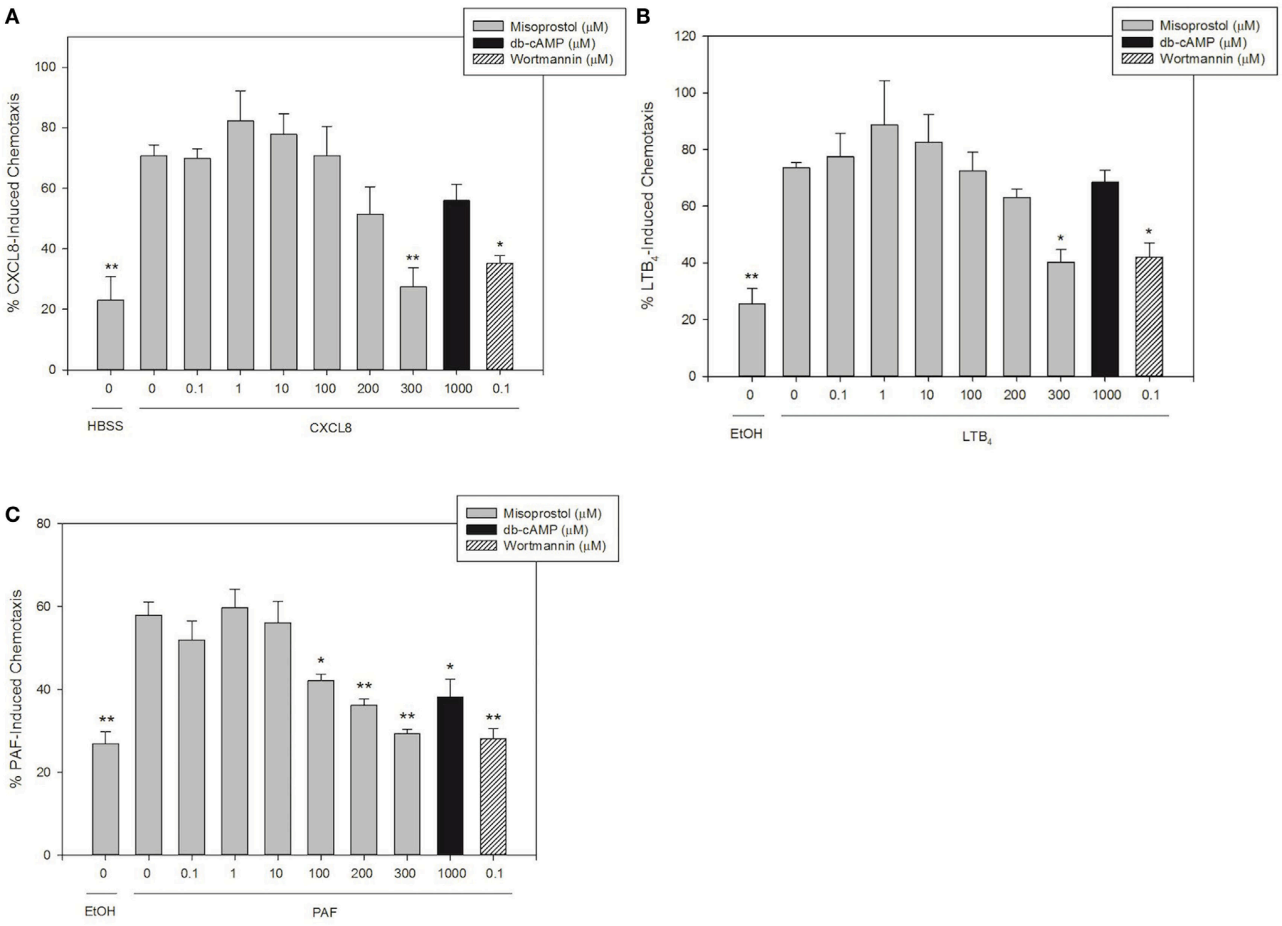
Misoprostol pretreatment inhibits CXCL8-, LTB_4_-, and PAF-induced equine neutrophil chemotaxis. Calcein AM-labeled equine neutrophils were pretreated with multiple concentrations of misoprostol, dibutyryl cyclic-AMP (db-cAMP), or the vehicle control (VC) for each inhibitor [Hank’s balanced salt solution (HBSS)]. The phosphatidylinositol-3 kinase (PI3K)-inhibitor wortmannin was used as a positive control for inhibition. Neutrophils were stimulated for 1 h with the following chemoattractants or vehicles: **(A)** 100 ng/ml CXCL8 (or HBSS vehicle), **(B)** 10 nM LTB_4_ (or EtOH vehicle), or **(C)** 10 nM PAF (or EtOH vehicle). Percent chemotaxis was calculated by dividing florescence in each bottom well by 100% migration control wells. Data are expressed as mean% chemotaxis ± SEM. **p < 0.001 and *p < 0.05 indicate significant difference from stimulated cells pretreated with the misoprostol vehicle *via* One-Way RM ANOVA; *n* = 3.

Concentrations of chemoattractants utilized for migration experiments were chosen based on previous work in our lab (27, 28, 30). LTB_4_ and CXCL8 were the most potent chemoattractants and induced directed migration of 73.6 and 70.8% of equine neutrophils, respectively (Figures 2A,B). While slightly less potent than LTB_4_ and CXCL8, PAF also induced significant neutrophil chemotaxis (58.0%, Figure 2C). Misoprostol significantly inhibited LTB_4_- and CXCL8-stimulated neutrophil migration at 300 µM (Figures 2A,B). Misoprostol significantly inhibited PAF-stimulated neutrophil migration at 100 µM (Figure 2C). Interestingly, db-cAMP significantly inhibited PAF-mediated neutrophil chemotaxis, but had no measured effect on LTB_4_- and CXCL8-induced migration (Figure 2).

### Neutrophil Production of ROS Is Increased by PMA and Immune Complexes in Unprimed Cells, and by LPS in GM-CSF Primed Cells

We defined the magnitude and kinetics of equine neutrophil ROS production using luminol-enhanced chemiluminesence to establish a reliable assay for the production and detection of equine neutrophil ROS production. Equine neutrophils were stimulated to produce ROS in response to IC, PMA, and LPS.

**Figure 3:**
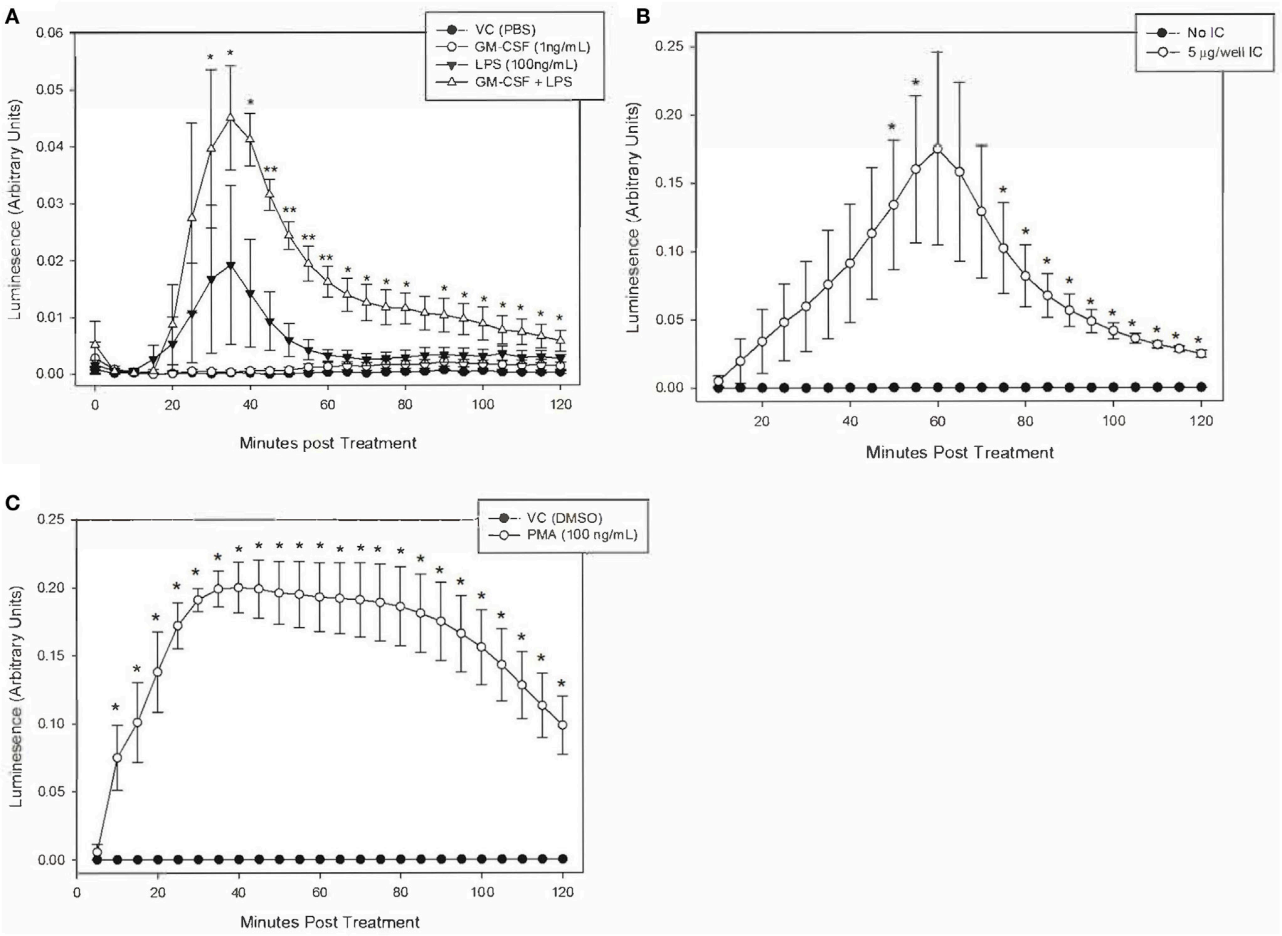
Kinetics of equine neutrophil reactive oxygen species production stimulated by lipopolysaccharide (LPS), immune complex (IC), and PMA. Equine neutrophils were treated with the following stimulants or vehicles: **(A)** 1 ng/ml GM-CSF priming for 30 min followed by stimulation with 100 ng/ml LPS (or PBS vehicle), **(B)** 5 µg immobilized IC (or 5% bovine serum albumin vehicle), or **(C)** 100 ng/ml PMA (or DMSO vehicle). Stimulated cells were incubated in the presence of 1 mM luminol for 2 h. Kinetic luminescence readings were taken every 5 min. Data are presented as mean ± SEM arbitrary units of luminescence. **p < 0.001 and *p < 0.05 indicate significant difference from unstimulated vehicle treated cells *via* One-Way RM ANOVA (LPS) or two-tailed *t*-test (IC and PMA); *n* = 3.

Equine neutrophils stimulated with 5 µg of immobilized IC produced a robust ROS response that peaked following 60 min of stimulation. This was followed by a decline in ROS production over the subsequent 60 min (Figure 3A). Because of high horse-to-horse variability, ROS production at this 60-min point was not considered significantly increased over unstimulated cells. However, the next highest point of ROS production at 55 min of IC stimulation was significantly different from controls (Figure 3A) and was, therefore, selected for subsequent experiments. PMA (100 ng/ml) stimulation resulted in significant ROS production by equine neutrophils at 10 min (Figure 3B).

Equine neutrophils stimulated with 100 ng/ml LPS showed a small increase in ROS production that was not significant compared to controls. Priming cells with 1 ng/ml GM-CSF for 30 min prior to LPS stimulation led to a significant increase in ROS production which peaked following 35 min of LPS treatment. GM-CSF priming alone did not have a significant effect on ROS production (Figure 3C).

It is worth noting that LPS treatment of primed equine neutrophils resulted in a less potent ROS response than PMA and IC. Peak luminescence values in these cells were one order of magnitude lower than those produced by IC and PMA treatment, indicating that LPS is a weaker stimulant of equine neutrophil ROS production, even following neutrophil priming (Figure 3).

### Misoprostol Pretreatment Significantly Inhibits IC-and LPS-Induced Equine Neutrophil ROS Production by Equine Neutrophils

**Figure 4:**
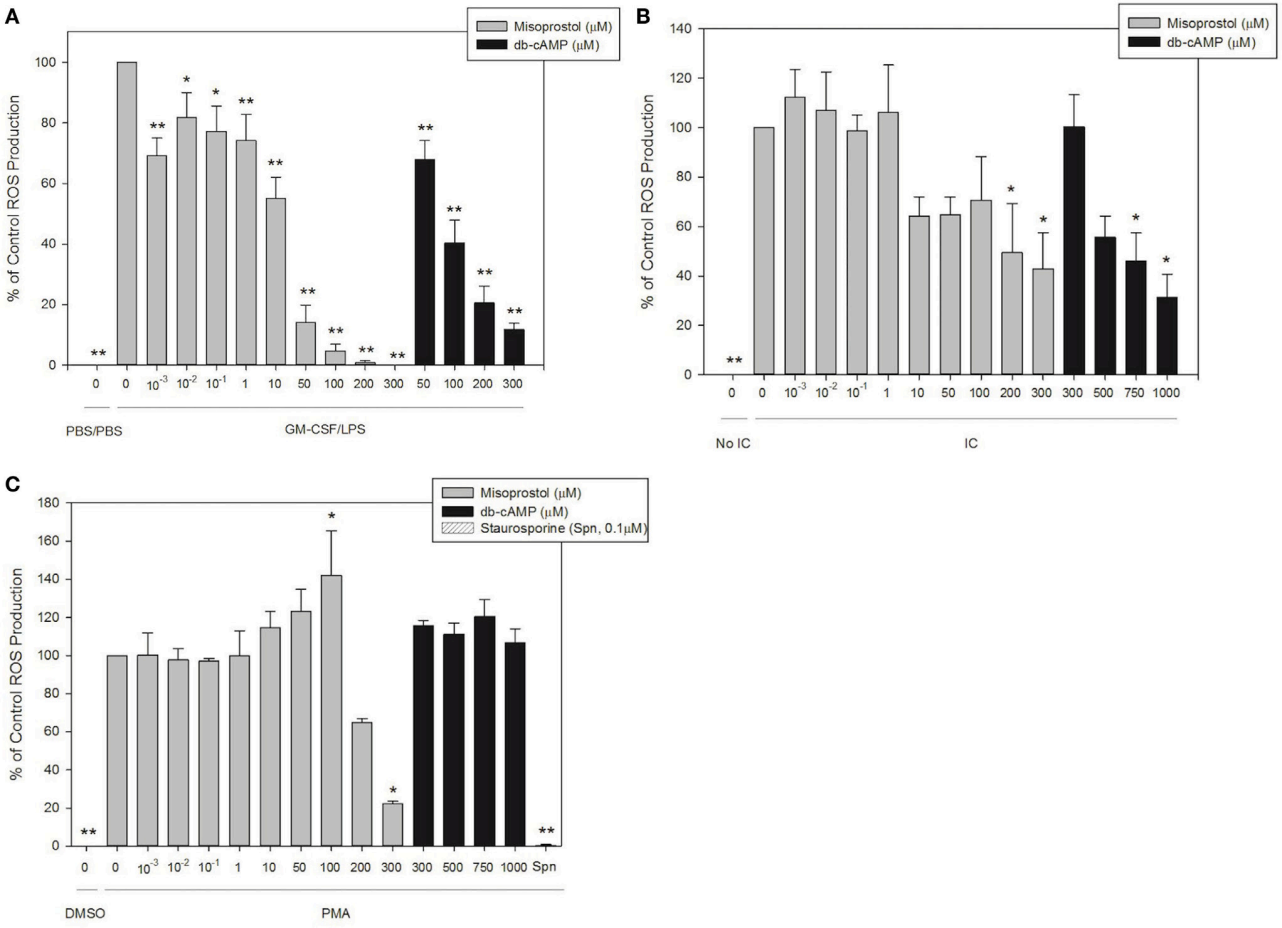
Pretreatment with misoprostol inhibits lipopolysaccharide (LPS)-, immune complex (IC)-, and PMA-stimulated equine neutrophil reactive oxygen species (ROS) production. Equine neutrophils were pretreated with multiple concentrations of misoprostol, dibutyryl cyclic-AMP (db-cAMP), or the vehicle control (VC) for each inhibitor [Hank’s balanced salt solution (IC and PMA), or PBS (LPS)]. The PKC inhibitor staurosporine was used as a positive control for inhibition of PMA-induced ROS production. Equine neutrophils were treated with **(A)** 1 ng/ml GM-CSF priming for 30 min followed by stimulation with LPS (or PBS vehicle) for 35 min; **(B)** 5 µg immobilized IC (or 5% fetal bovine serum) for 55 min; or **(C)** 100 ng/ml PMA (or DMSO vehicle) for 40 min, in the presence of 1 mM luminol. Data are presented as mean% ROS production ± SEM normalized to stimulated cells pretreated with the misoprostol vehicle. **p < 0.001 and *p < 0.05 indicate significant difference from stimulated cells pretreated with the misoprostol vehicle *via* One-Way RM ANOVA; *n* = 5 (GM-CSF/LPS), *n* = 3 (IC and PMA).

We next utilized our validated luminol-enhanced chemiluminescence assay to evaluate the effect of misoprostol on ROS production by isolated equine peripheral blood neutrophils in response to our three stimulants, IC, PMA and LPS. Misoprostol pretreatment of 200 and 300 µM inhibited IC-stimulated ROS production in a concentration-dependent manner to 49.4 and 42.9% of control, respectively (Figure 4A). db-cAMP pretreatment also inhibited IC-stimulated ROS production in a concentration-dependent manner at 750 µM and higher (Figure 4A).

Most concentrations of misoprostol pretreatment actually enhanced PMA-stimulated ROS production of equine neutrophils in a concentration-dependent manner (Figure 4B). Unlike the other concentrations tested, 300 µM misoprostol pretreatment significantly inhibited PMA-mediated neutrophil ROS production (Figure 4B). db-cAMP had no effect on PMA-stimulated ROS production. Based on the db-cAMP treatment results, we suspect the inhibitory effect seen with 300 µM misoprostol might be attributed to cAMP-independent mechanisms. The PKC inhibitor staurosporine was used as a positive control for ROS inhibition (Figure 4B).

Misoprostol pretreatment significantly inhibited LPS-stimulated ROS production in primed equine neutrophils. This effect was concentration dependent, and was observed even at even the lowest misoprostol concentration. Similarly, db-cAMP pretreatment inhibited ROS production in a concentration-dependent manner (Figure 4C).

## Discussion

The aim of this study was to investigate the hypothesis that the cAMP-elevating agent misoprostol would have anti-inflammatory effects on equine neutrophil functions, including adhesion, chemotaxis, and ROS production. Consistent with our hypothesis, we show that misoprostol pretreatment inhibits LTB_4_-stimulated adhesion (Figure 1A), LTB_4_-, CXCL8-, and PAF-stimulated migration (Figure 2), and LPS-, IC- and PMA-stimulated ROS generation (Figure 4) of isolated, primary equine neutrophils. Misoprostol pretreatment had no effect on IC-induced adhesion and actually increased adhesion of neutrophils treated with PMA (Figures 1B,C). This study brings to light important information about misoprostol, which is a clinically relevant therapeutic that has been widely used to treat horses with colonic ulceration and has more recently been reported to be superior to omeprazole and sucralfate for healing gastric glandular lesions in horses with clinical disease (31). To our knowledge, we are the first group to establish anti-inflammatory effects of misoprostol on equine neutrophil functions *in vitro*. Given the recent report that misoprostol is an excellent therapeutic for healing gastric glandular lesions in horses, our *in vitro* results offer relevant information regarding the anti-inflammatory effects of misoprostol.

The presumed mechanism for misoprostol’s anti-inflammatory effects on neutrophil functions is EP2 and EP4 receptor binding. Activation of these receptors is known to increase intracellular cAMP (15, 19, 21). cAMP is a ubiquitously produced second messenger molecule that is generated through GPCR signaling in neutrophils. Ligand binding to GPCRs leads to activation of intracellular AC, which catalyzes the cyclization of AMP to form cAMP. cAMP activates two different intracellular pathways that mediate neutrophil adhesion, protein kinase A (PKA) and exchange proteins directly activated by cAMP (Epac) (10, 11). Interestingly, the two pathways activated by cAMP have been shown to induce opposing cell signaling cascades. While PKA signaling in neutrophils is primarily inhibitory, Epac signaling is associated with neutrophil activation (32). Recently, it has been suggested that PKA is the predominant cAMP pathway within neutrophils, and thus agents that elevate cAMP hold great promise as inhibitors of neutrophil function in inflammatory disease (33).

Previous reports demonstrate a direct link between elevation of intracellular cAMP and inhibition of equine neutrophil functions. Similarities between the effects of misoprostol and db-cAMP in our system suggest that increased cAMP is the predominant mechanism through which misoprostol inhibits most of the evaluated equine neutrophil functions. However, as we did not measure cAMP levels in response to misoprostol pretreatment, additional studies are needed to determine if there is a direct link between misoprostol, intracellular cAMP, and neutrophil functions.

To enter inflamed tissues, circulating peripheral blood neutrophils must first adhere to endothelial cells neighboring injured tissues. This requires transient chemoattractant-induced adhesion, followed by firmer adhesion of activated neutrophils. Both adhesion events are mediated by β_2_ integrins (34). Increased intracellular cAMP has been shown to inhibit β_2_ integrin-dependent adherence of equine neutrophils (10, 11). In this study, pretreatment of equine neutrophils with misoprostol, a cAMP-elevating agent, inhibited LTB_4_- and IC-induced adhesion in a concentration-dependent manner. However, in contrast to previous studies in our lab (11), inhibition only achieved statistical significance in LTB_4_ but not IC-stimulated cells (Figures 1A,B). We hypothesize that divergent signaling pathways induced by these endogenous stimulants lead to differing effects of misoprostol on transient (LTB_4_), versus firm (IC), neutrophil adhesion. In human neutrophils, LTB_4_-mediated GPCR-stimulated adhesion is PI3K-independent, while IC-mediated FcγR-stimulated adhesion is PI3K dependent (35). These findings are supported in this study in equine neutrophils (Figures 1A,B). Differences in PI3K dependence, as well as additional downstream signaling molecules, could explain the weaker inhibitory effects of misoprostol on IC- versus LTB_4_-stimulated equine neutrophil adhesion.

In contrast to LTB_4_ and IC, misoprostol pretreatment led to a dose-dependent increase in PMA-stimulated adhesion that is similar to previous reports from our lab (11). PMA is a synthetic mimic of diacylglycerol and permeates the cell to directly activate PKC. Because PMA bypasses cell surface receptor activation it is a non-physiologic stimulus of neutrophil adhesion (1). From this experiment, we conclude that misoprostol inhibits equine neutrophil adhesion responses through a mechanism that is upstream of PKC-activation.

Following adhesion to the vascular endothelium, neutrophils must crawl along the vascular endothelium and undergo directed interstitial tissue migration in response to chemoattractant gradients to reach sites of tissue injury or infection (36, 37). Chemoattractants induce neutrophil migration by engaging GPCRs and activating many downstream signaling pathways, including mitogen-activated protein kinases, phospholipase C, and PI3K. While many of these mechanisms are shared, each unique GPCR initiates different chemotactic responses, intensities, and migration patterns (38). Lipid chemoattractants (such as LTB_4_ and PAF) *initiate* neutrophil chemotaxis into inflamed tissues, while chemokines such as CXCL8 act at *later* stages to amplify neutrophil chemotaxis (39). Because of these differences, we evaluated the effect of misoprostol on multiple types of chemoattractants including the lipids LTB_4_ and PAF, and the chemokine CXCL8.

Human neutrophil migration is enhanced by 1 µM PGE_1_ pretreatment in response to fMLP, but is inhibited at higher PGE_1_ concentrations (40). In this study, misoprostol pretreatment inhibited equine neutrophil chemotaxis toward CXCL8, LTB_4_, and PAF (Figure 2). Misoprostol most potently inhibited chemotaxis toward PAF, which was the weakest chemoattractant evaluated (Figure 2C). With the more potent chemoattractants (LTB_4_ and CXCL8), 300 µM concentration of misoprostol was significantly inhibitory. Interestingly, 1–10 µM misoprostol showed a trend toward enhancing LTB_4_- and CXCL8-directed migration, but this was not statistically significant (Figures 2A,B). These data are consistent with previous reports that low versus high levels of cAMP can stimulate or inhibit neutrophil migration, respectively (41). Previous studies have demonstrated that endogenous PGEs inhibit cell migration through an EP2 receptor mediated increase in intracellular cAMP (12, 15, 21). Additionally, cAMP’s effect on neutrophil chemotaxis varies depending on the concentration of the chemoattractant. For example, increased intracellular cAMP in the presence of optimal concentrations of LTB_4_ has little effect on neutrophil migration; but the same levels of intracellular cAMP significantly inhibit neutrophil migration toward threshold concentrations of LTB_4_ (defined as the lowest concentrations of LTB_4_ that elicit a significant chemotactic response) (12). Taken together with our findings, it is likely that the effects of cAMP on equine neutrophil chemotaxis are dependent on the specific cAMP-elevating agent and the chemoattractant concentration utilized.

Once within tissues, neutrophils are designed to produce ROS such as such as superoxide $\left(O_{2}^{-}\right)$ and hydrogen peroxide (H_2_O_2_) to kill bacterial pathogens. Neutrophils are capable of releasing ROS intracellularly within phagosomes containing engulfed microbes, as well as into the surrounding tissues to kill nearby pathogens. Release of ROS into surrounding tissues can significantly contribute to host tissue damage in many disease states; in patients with inflammatory, rather than infectious diseases, overabundant ROS production can cause substantial tissue injury. Equine patients with inflammatory diseases would benefit from therapies that restrict neutrophil ROS production. Misoprostol and other cAMP-elevating agents are known to inhibit human neutrophil ROS production (19, 42). Therefore, we hypothesized that misoprostol would inhibit ROS production by LPS-stimulated equine neutrophils. To detect both intra- and extra-cellular neutrophil ROS production, both of which can contribute to host tissue damage, we utilized luminol-enhanced chemiluminescence methods (43).

Because LPS alone induced minimal ROS production in equine neutrophils, we hypothesized that priming neutrophils with granulocyte-monocyte colony-stimulating factor (GM-CSF) would enhance LPS stimulation. This hypothesis was based on previous studies which showed that neutrophil priming with GM-CSF prior to stimulation led to more robust ROS generation (44). Consistent with this report, we show that GM-CSF-primed, LPS-stimulated equine neutrophils generate significantly higher levels of ROS compared to LPS-stimulated cells that have not been primed (Figure 3C).

Misoprostol and db-cAMP inhibited IC and LPS-stimulated ROS production in a concentration-dependent manner in our study (Figures 4A,C). IC- and LPS-stimulated ROS production is dependent on PI3K activation and is inhibited by PKA (45, 46). Together, these data suggest that misoprostol likely inhibits ROS production in IC and LPS-stimulated neutrophils through cAMP-activated PKA. In contrast, PMA stimulates ROS production through direct activation of PKC (47, 48). Therefore, this pathway is generally thought to be insensitive to cAMP-elevating agents (42, 45). Interestingly, while db-cAMP had no effect on PMA-mediated ROS production, 100 µM misoprostol significantly increased ROS levels. Conversely, 300 µM misoprostol significantly decreased ROS production (Figure 4B), which is consistent with previously published findings (49). Insensitivity of PMA-induced ROS generation to cAMP helps to explain these contradictory findings.

Misoprostol is currently used to treat and prevent NSAID-induced GI injury in equine patients suffering from inflammatory disease (23, 24). While select non-steroidal anti-inflammatory drug have been shown to inhibit neutrophil adhesion (50), chemotaxis (51), and respiratory burst (52), previous reports suggest these effects are further augmented by misoprostol (53, 54). Previous research has also shown that misoprostol restores mucosal barrier function following ischemia-reperfusion injury in equine small intestine, potentially through a cAMP-dependent mechanism (26, 55). Therefore, the addition of misoprostol to NSAID therapy could lead to more complete inhibition of neutrophilic inflammation with fewer GI side effects compared to NSAID treatment alone. Based on these previous reports and our current data, we propose that combining misoprostol with NSAID therapy in horses could help maintain GI health and could potentially inhibit neutrophil inflammatory functions.

This study demonstrates for the first time that misoprostol exerts anti-inflammatory effects on equine neutrophil effector functions *in vitro*. While it is true that relatively high concentrations of misoprostol were necessary for significant inhibition of neutrophil function *in vitro*, this does not preclude our data from being clinically relevant. We propose that orally administered misoprostol may produce local, cAMP-mediated, anti-inflammatory effects on injured GI mucosa. While these data are promising, high doses of misoprostol have been associated with negative side effect in human, canine, and equine patients, which include abdominal discomfort and diarrhea (56, 57). Additional studies utilizing e*x- and in vivo* equine inflammatory models are currently underway to investigate this hypothesis and assess the safety profile of this drug in relation to potential anti-inflammatory effects. Additionally, these data provide proof of principle that misoprostol, a known EP receptor agonist, elicits anti-inflammatory effects on equine neutrophils. Based on this finding, we are currently conducting additional studies to evaluate the anti-inflammatory effects of specific EP2 and/or EP4 receptor agonists on equine leukocytes.

## Ethics Statement

This study was approved by the Institutional Animal Care and Use Committee (IACUC) at North Carolina State University.

## Author Contributions

EM was responsible for study design, experimental execution, and preparing the manuscript. RT performed all neutrophil adhesion experiments and critically reviewed the manuscript. MS critically reviewed the manuscript and aided in figure design and layout. SJ was responsible for overseeing all aspects of the study, including study design and critically reviewing the manuscript.

## Conflict of Interest Statement

This research was conducted in the absence of any commercial or financial conflicts of interest.
